# Cannabis and amphetamine use among school-going adolescents in sub-Saharan Africa: a multi-country analysis of prevalence and associated factors

**DOI:** 10.1186/s12888-023-05283-w

**Published:** 2023-10-24

**Authors:** Kwaku Oppong Asante, Prince Atorkey

**Affiliations:** 1 Department of Psychology, School of Social Sciences, College of Humanities, University of Ghana, P.O. Box LG 84, Legon, Accra Ghana; 2 Department of Psychology, University of the Free State, Bloemfontein, South Africa; 3 Discipline of Psychological Sciences, Australian College of Applied Professions, Sydney, Australia; 4 School of Medicine and Public Health, The University of Newcastle, Callaghan, Australia; 5 Hunter Medical Research Institute, New Lambton Heights, NSW 2305 Australia

**Keywords:** School-going adolescents; amphetamine use, Cannabis use, Sub-saharan Africa

## Abstract

**Background:**

Global evidence indicates that early onset of illicit substance use among adolescents and emerging adults is associated with negative mental-health related-outcomes that can persist into adulthood. However, the lack of quality regional data on adolescent illicit substance use and its determinants remains a common barrier to evidence-based policy-making and the development of school-based interventions in Africa. The purpose of our study was to estimate the prevalence and describe the correlates of cannabis and amphetamine use among school-going adolescents in eight sub-Saharan African countries (SSA) – Benin, Ghana, Liberia, Mauritius, Mozambique, Namibia, Seychelles, and Tanzania.

**Methods:**

We analysed 15,553 school-going adolescents that participated in the Global School-based Student Health Survey. A two-stage sampling approach was used to generate a nationally representative sample of school children (grades 7–12) in each of these countries. Students responded to a self-administered structured questionnaire that contained information on sociodemographic factors, family involvement factors, mental health factors, school environment factors and past-month cannabis and life-time amphetamine use.

**Results:**

The overall prevalence estimates of past-month cannabis use and lifetime amphetamine use among school-going adolescents in the eight SSA countries was 4.39% (95% CI = 4.08, 4.72) and 3.05% (95% CI = 2.79, 3.34) respectively. In the multivariable logistic regression analysis, demographic characteristics (age and male gender), mental health factors (suicide ideation and attempt), lifestyle factors (cigarette smoking, past-month alcohol use, lifetime drunkenness and leisure-time sedentary behaviour) and school level factors (truancy and bullying victimisation) showed strong associations with increased odds of both past-month cannabis use and lifetime amphetamine use. Social support at school was associated with increased odds for lifetime amphetamine, while parental monitoring decreases the odds for lifetime amphetamine use. It was also observed that parental tobacco use was associated with increased odds of both past-month cannabis use and lifetime amphetamine use.

**Conclusion:**

The relatively low overall prevalence estimates of past-month cannabis use and lifetime amphetamine use among school-going adolescents in not surprising. However, the identified risk and protective factors associated with cannabis and amphetamine use underscores the need for these eight countries in SSA to develop contextual and multi-sectoral intervention and school-based prevention programmes that could target school-going adolescents who may be at risk of misusing these illicit drugs.

**Supplementary Information:**

The online version contains supplementary material available at 10.1186/s12888-023-05283-w.

## Background

The United Nations Office on Drugs and Crime (UNODC) has indicated that there is an increase of global prevalence of illicit drug use (including cannabis and amphetamine) for the past decade (2010–2022) [1]. The same report revealed that amphetamine, cannabis, cocaine, and opioids were the most frequently used illicit drugs [1]. Approximately 209 million and 34 million people as at 2020 had used cannabis and amphetamine respectively [1]. In sub-Saharan Africa (SSA) for example, 2.7 million people were reported to be users of amphetamine, out of which 780,000 were from West and Central Africa. Within the same SSA, the 12-month prevalence of cannabis use in 2020 is higher than the global average estimated at 6.5% of the population aged 15–64, with West and Central Africa having the highest prevalence use within the sub-region [1]. This high prevalence of illicit drugs use is a public mental health problem as it contributes to the global burden of diseases [2]. Indeed, evidence from the Economic Community of West African States (ECOWAS) indicates that half of all people treated for drug use disorders in Africa in 2020 were treated for cannabis as the primary drug of concern (far higher proportion than in any other region [3].

Adolescent use of illicit drugs affects their health and wellbeing; and is associated with negative outcomes such as mental health problems and neurocognitive impairments that can persist into adulthood [2]. This use of illicit substances typically occurs during adolescence, as it is often associated with experimentation and to deal with the transition from childhood to adolescence [4]; and adolescents are usually susceptible to the adverse effects of illicit drug use during this developmental stage [2, 5]. Evidence from Western countries have also indicated that individuals who use cannabis and amphetamine during adolescence are at elevated risk to develop cannabis use disorder during adulthood [6]. Despite these evidences from high resourced countries, there is lack of regional data on illicit substance use (particularly cannabis and amphetamine use) that affects the development of policy and school-based interventions within sub-Saharan Africa (SSA). In SSA, only few studies have explored the prevalence of cannabis and amphetamine use among in-school adolescents with prevalence of 5.6-7.1% for lifetime amphetamine and 4-5.3% reported past-month cannabis use [7–9]. A recent systematic review has suggested the need for more studies to be conducted on young adult’s cannabis and amphetamine use, as there is lack of data on such populations within the African context [10].

Existing scholarly evidence within SSA and other low - and middle-income countries (LAMICs), showed that there are multifaceted factors that are associated with both cannabis and amphetamine use among adolescents and young adults. We identified these factors to exist at the personal/individual level such as gender, age, and grade [11–13], mental health-related factor such as loneliness, anxiety, and suicidal behaviour [12, 14, 15] and lifestyle factors (mainly, health risk behaviours) such as cigarette smoking and leisure-time sedentary behaviour [7, 12, 13, 16]. Additionally, school-level factors found to be associated with alcohol use are truancy and bullying victimisation at school [7, 13] and interpersonal factors implicated in both cannabis and amphetamine use included higher number of friends, and frequent involvement in a physical fight [7, 17]. Finally, within the family environment, parental monitoring, parental understanding, parental supervision, parental intrusion of privacy, and parental tobacco use have been found to be associated with alcohol use and drunkenness among school-attending adolescents [7, 12, 18, 19].

The factors that influence both cannabis and amphetamine use exist at various levels. Thus, our study is located within an adapted socio-ecological theory [20]. The socio-ecological model is a framework for prevention and considers the complex inter-relationship between individual, interpersonal, community and societal factors. Fundamentally, this model recognises the importance of a multi-layered environment impacting mental health and wellbeing. Thus, in understanding adolescent substance use, we need to consider the different levels of influence not only at an individual level, but also at the immediate and the broader community and society levels as well. In applying this framework to this study, the importance of adolescents’ personal, interpersonal relationships, and parental factors, and school environmental factors, cannot be overemphasized, as they have been found to influence their substance use behaviour including cannabis and amphetamine use [2, 20]. Notwithstanding the above reviewed literature, to the best of the authors’ knowledge we are not aware of any available study that have examined the prevalence and determinants of both cannabis and amphetamine among school-going adolescents using a pooled data from eight sub-Saharan African countries.

In order to develop interventions that target school-going adolescents that may be at risk for regular cannabis and lifetime amphetamine use, a nationally representative data is needed to help address the mental health needs of adolescents and young adults within the sub-region. Our findings will help countries within SSA towards the attainment of the Sustainable Development Goal (SDGs) 3, target 3.5 that seeks to strengthen the prevention and treatment of substance abuse, including narcotic drug abuse and harmful use of alcohol. In order to fill the gap in scientific discourse, this study was conducted to examine the prevalence and determinants of both cannabis and amphetamine among school-going adolescents in SSA using data from eight countries that participated in the World Health Organisation’s (WHO) Global School-based Health Survey (GSHS). This study focused on past-month cannabis use and lifetime amphetamine use because regular cannabis use is more common among this population than lifetime amphetamine use due to its less accessibility among the same population [1, 2]. Based on the theoretical framework, the following two key objectives were examined: (1) to estimate the prevalence of past-month cannabis use and lifetime amphetamine use among school-going adolescents in eight SSA countries; and (2) to explore individual, mental health, lifestyle, family-level, school-related and interpersonal factors that are associated with past past-month cannabis use and lifetime amphetamine use among school-going adolescents in eight SSA countries.

## Methods

### Study design

This study adopted a cross-sectional design using secondary data from the Global School Health Survey (GSHS) conducted between 2012 and 2017 from eight countries in sub-Saharan Africa. This study has been reported according to Strengthening the Reporting of Observational Studies in Epidemiology (STROBE) criteria [21].

### Study context

This study pooled data on school-going adolescents in eight sub-Saharan African countries (SSA) – Benin, Ghana, Liberia, Mauritius, Mozambique, Namibia, Seychelles, and Tanzania. We herein provide some background information on these countries. Benin is a Francophone country of 11.5 million people, with 32.6% of the population aged less than 25 years [22, 23], with a low human development index (HDI rank of 163), mean years of schooling of 3.8 years, and life expectancy of 61.5 years in 2018 [24]. It is considered a low-income country [25] with a secondary school enrollment rate of 47.8%. Ghana is an Anglophone country estimated to be inhabited by 30.9 million people, with 31.6% of the population aged 10–24 years [23, 26]. It is categorised as having a medium human development index (HDI rank of 176), with a life expectancy of 61.5 years in 2018, and mean years of schooling of 4.7 years [24]. Ghana is a lower-middle income country [25]. It has a secondary school enrollment rate of 77.7%. Liberia is an Anglophone country categorised as a low-income country, with a low human development index [HDI rank of 176] [24, 25]; and has a population of about 4.8 million people, with the population structure described as young; 63% is less than 25 years old and 32.8% is 10–24 years old [22, 23]. Life expectancy in the country is 63.7 years, with mean years of schooling of 4.7 years [24], and secondary school enrollment rate of 77.7%.

Mauritius is categorised as an upper middle-income country, with a high human development index [HDI rank of 63] [24, 25]; and has a population of about 1.3 million people, with the population structure described as young; 71% are between the ages of 15–64 years old [22]. Life expectancy in the country is 74 years, with mean years of schooling of 10.4 years [24] and secondary school enrollment rate of 93.7%. Mozambique is categorised as a low-income country, with a high human development index [HDI rank of 185)] [24, 25]; and has a population of about 32.9 million people, with over half of the population (51.5%) between the ages of 15–64 years old [22]. Life expectancy in the country is 59 years, with mean years of schooling of 7.6 years [24] and secondary school enrollment rate of 38.8%. Namibia has a youthful population, as persons aged 17 years and younger constitute 43% of the general population [22]. The mean years of schooling in Namibia is 7.2 years [24]. Primary to middle school education, which stretches from grade 1 through 7, is compulsory for all children between the ages of 6 and 16 years, while secondary education remains optional, but it’s school enrollment rate of 65.8% [22]. Namibia is an English-speaking Southern sub-Saharan African country classified as an upper-middle-income country [24], with a Medium Human Development Index rank of 139 [24, 25].

Seychelles is categorised as a high-income country, with a high human development index [HDI rank of 72] [24, 25]; and has a population of about 100,000 people. It has a youthful population, as persons aged 24 years and younger constitute 51.7% of the general population [22]. Life expectancy in the country is 73 years, with mean years of schooling of 13.1 years [24] and secondary school enrollment rate of 78.2%. Tanzania is categorised as a lower-income country, with a high human development index [HDI rank of 160] [24, 25]; and has a population of about 65.5 million people. Approximately 44% of Tanzania’s population was under age 15 years [22]. Life expectancy in the country is 66 years, with mean years of schooling of 6.5 years [24] and secondary school enrollment rate of 28.7%.

### Participants and procedure

**Fig. 1:**
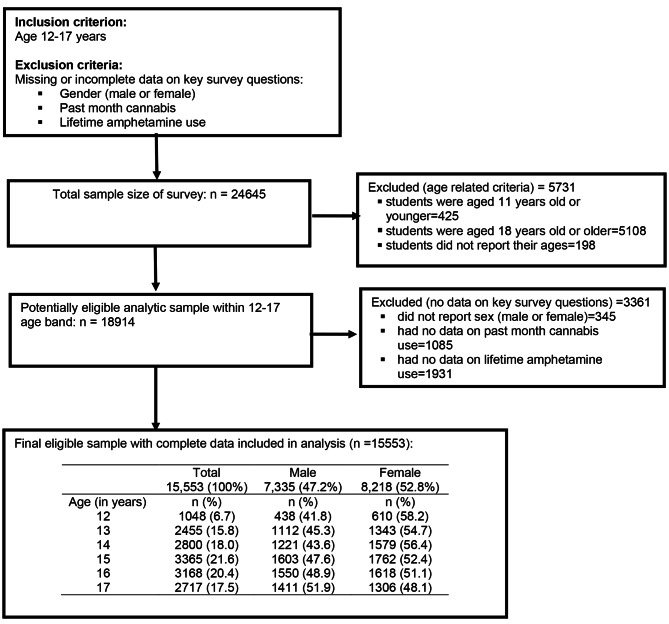
Flow diagram of analytic sample selection process and criteria

Participants in this study were school-going adolescents from eight sub-Saharan African countries. These countries included Benin (2016), Ghana (2012), Liberia (2017), Mauritius (2017), Mozambique (2015), Namibia (2013), Seychelles (2015), and Tanzania (2014). Data was obtained from a nationally representative sample of secondary school students who completed a computer-scannable questionnaire during their class period. A two-stage cluster sample design was used to produce data representative of all students in grades 7–12 within these participating countries. At the first stage, schools were selected with probability proportional to enrollment size. At the second stage, classes were randomly selected and all students in selected classes were eligible to participate. As stipulated by WHO-GSHS, participation in the study was voluntary, anonymous, and confidential as no identifying information was obtained [27]. To get the analytical sample, we applied inclusion and exclusion criteria shown in Fig. 1. The total sample of students that participated in the survey was 24,645. However, after the inclusion and exclusion criteria were applied, the final sample size included in this study was 15,553. This constitutes 7,335 (47.2%) who identified as males and 8,218 (52.8%) who identified as females with an average age of 14.9 years (*SD* = 1.52).

### Measures

#### Outcome variables

This study had two outcome variables (i.e., cannabis use and amphetamine use). Cannabis use was measured using a single item (i.e., “During the past 30 days, how many times have you used marijuana”). Similarly, amphetamine use was measured using a single item (i.e., During your life, how many times have you used amphetamine or methamphetamine (also called ice or yellow?”). Further details (i.e., the questions used in measuring each variable, response options, and coding of responses for analysis) about all variables included in this study can be found in the Supplementary e-Table 1.

#### Exposure variables

The exposure variables in this study included: *mental health factors* (i.e., anxiety, loneliness, suicidal ideation, suicidal attempt); *lifestyle factors* (i.e., cigarette smoking, past-month alcohol use, lifetime drunkenness, leisure-time sedentary behaviour); *school-level factors* (i.e., truancy, bullying victimization, social support at school); *interpersonal-level factors* (number of close friends, physical fight); and *family-level factors* (i.e., parental supervision, parental understanding, parental monitoring, parental intrusion of privacy, parental tobacco use). The inclusion of these variables were based on previous publications based on the WHO-GSHS data [7, 28–30]. The questions used in assessing each variable, the original responses, and how these responses were recoded for statistical analysis are presented in Supplementary e-Table 1.

#### Demographic variables

Gender (male or female) and age were demographic variables included in this study.

### Statistical analyses

Due to the nature of the study design, numerical weighting was applied to each respondent record to enable generalisation of results to study population. This included using the primary sampling units (PSU) and strata at the country-specific data. Statistical analysis was carried out in three stages using STATA software version 17.0 (Stata Corporation, College Station, TX, USA). Firstly, a univariate analysis (i.e., percentages and frequencies) to determine prevalence of cannabis use and amphetamine use. The second stage involved a bivariate analysis using chi-square (χ^2^) to examine the relationship between the exposure variables and the outcome variables. Finally, a multivariate binomial logistic regression was performed to determine which exposure variables were associated with each of the outcome variables. Results of the univariate analysis are presented in a graphical form and results of the multivariate binomial logistic regression are reported using adjusted odd ratios (AOR) with their corresponding 95% confidence intervals (95% CI) in tabular form. Using the missing data analysis theory [31, 32], the list-wise deletion approach was used to handle missing data, as predominately, the missing data were less than 5%. Statistical significance was defined as a two-tailed *p*-value < 0.05 in all analyses.

### Ethical considerations

Given that the dataset used for this study was secondary data, the authors did not seek ethical clearance. However, prior to the gathering of data by the GSHS team, ethical approval was sought from the World Health Organisation, Centre for Disease Control and Middle Tennessee State University and the participating countries Ministries/Agencies in charge of Education and Health. All ethical guidelines concerning the use of human subjects, especially minors, were strictly adhered to. Written parental/guardian consent and assent from children under 18 years was obtained. The sampled students anonymously and voluntarily completed the survey questionnaire. As this study used secondary data, ethical clearance was waived by the Departmental Research and Ethics Committee (DREC) and the Ethics Committee of the Humanities (ECH), all from the University of Ghana.

## Results

### Prevalence of past-month cannabis and lifetime amphetamine among school-going adolescents

**Fig. 2:**
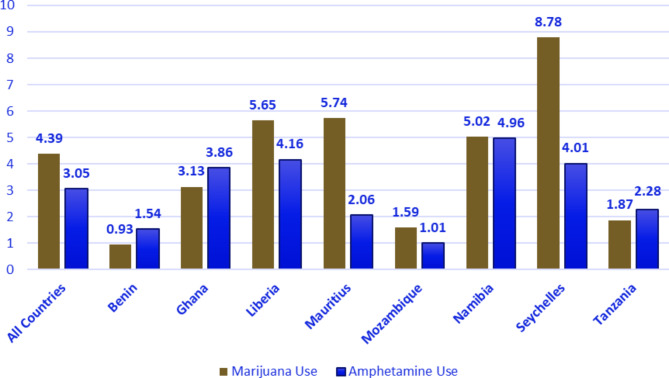
Prevalence of cannabis use and amphetamine use among in-school adolescents

The overall prevalence of cannabis use and amphetamine use among school-going adolescents in SSA was 4.39% (95% CI = 4.08, 4.72) and 3.05% (95% CI = 2.79, 3.34) respectively. At the country level, Seychelles had the highest level of marijuana use [8.78% (95% CI = 7.67, 10.02)], followed by Mauritius [5.74% (95% CI = 4.94, 6.66)] and Liberia [5.65% (95% CI = 4.38, 7.25)]. School going adolescents in Namibia reported the highest prevalence of amphetamine use [4.96% (95% CI = 4.24, 5.79)], followed by Liberia [4.16% (95% CI = 3.09, 5.59)] and Seychelles [4.01% (95% CI = 3.27, 4.90]. This information are presented in Fig. 2 below.

### Bivariate analysis of the factors associated with cannabis and amphetamine use

The relationship between the past-month cannabis use, lifetime amphetamine use, and other explanatory variables are presented in Table 1. We found that mental health factors (loneliness, suicidal ideation, and attempt), lifestyle factors (cigarette smoking, past-month alcohol use, lifetime drunkenness and leisure-time sedentary behaviours), school-level factors (truancy and bullying victimisation) and interpersonal level factors (number of close friends and engagement in physical fight) were all significantly associated with both past-month cannabis use and lifetime amphetamine. For the family-level factors, while parental understanding, monitoring, intrusion of privacy and parental tobacco use were significantly associated with both past-month cannabis use and lifetime amphetamine use, parental supervision was associated with only past-month cannabis use. Social support at school was only associated with lifetime amphetamine use. In terms of gender, the prevalence estimate was significantly higher among males than females for both past-month cannabis use (${\chi ^2}$= 115.15, *p* < 0.001) and lifetime amphetamine (${\chi ^2}$= 42.68, *p* < 0.001).

**Table 1:** Bivariate analysis of the factors associated with cannabis and amphetamine use

Variables		Weighted *N*	Weighted %	Past-month cannabis use		Lifetime amphetamine use	
				Yes (%)	*p-values*	Yes (%)	*p-values*
***Demographic***							
**Gender**					< 0.001		< 0.001
	Male	7335	47.2	459 (6.3)		294 (4.01)	
	Female	8218	52.8	224 (2.7)		181 (2.2)	
**Mental health factors**							
	**Loneliness**				< 0.001		< 0.001
	Yes	8809	58.8	457 (5.1)		313 (3.5)	
	No	6542	41.2	209 (3.3)		145 (2.3)	
	**Anxiety**				< 0.001		< 0.001
	Yes	8809	57.4	477 (5.4)		346 (3.9)	
	No	6542	42.6	183 (2.8)		106 (1.6)	
	**Suicidal ideation**				< 0.001		< 0.001
	Yes	2533	16.7	195 (7.7)		161 (6.4)	
	No	12,673	83.3	410 (3.2)		243 (1.9)	
	**Suicidal attempt**				< 0.001		< 0.001
	Yes	2485	16.2	278 (11.2)		261 (10.5)	
	No	12,828	83.8	379 (3.0)		183 (1.4)	
**Lifestyle factors**							
	**Cigarette smoking**				< 0.001		< 0.001
	Yes	1319	8.6	404 (30.6)		222 (16.8)	
	No	13,960	91.4	220 (1.6)		202 (1.5)	
	**Past-month alcohol use**				< 0.001		< 0.001
	Yes	3460	23.4	458 (13.2)		265 (7.7)	
	No	11,322	76.6	156 (1.4)		135 (1.2)	
	**Lifetime drunkenness**				< 0.001		< 0.001
	Yes	2811	18.7	418 (14.9)		237 (8.4)	
	No	12,203	81.3	167 (1.4)		136 (1.1)	
	**Leisure-time sedentary behaviour**				< 0.001		< 0.001
	Yes	5037	33.0	356 (7.1)		223 (4.4)	
	No	10,209	67.0	291 (2.9)		221 (2.2)	
**School-level factors**							
	**Truancy**				< 0.001		< 0.001
	Yes	3767	24.7	392 (10.4)		275 (7.3)	
	No	11,489	75.3	250 (2.2)		160 (1.4)	
	**Bullying victimization**				< 0.001		< 0.001
	Yes	5224	36.3	347 (6.6)		292 (5.6)	
	No	9194	63.7	262 (2.9)		117 (1.3)	
	**Social support at school**				0.275		0.009
	Yes	12,211	79.9	518 (4.2)		333 (2.7)	
	No	3070	20.1	144 (4.7)		123 (4.0)	
**Interpersonal-level factors**							
	**Number of close friends**				< 0.001		< 0.001
	Yes	13,707	10.3	546 (4.0)		346 (2.5)	
	No	1589	89.6	92 (5.8)		89 (5.6)	
	**Physical fight**				< 0.001		< 0.001
	Yes	4594	29.8	406 (8.8)		304 (6.6)	
	No	10,830	70.2	265 (2.5)		163 (1.5)	
**Family-level factors**							
	**Parental supervision**				< 0.001		0.084
	Yes	11,750	76.9	452 (3.9)		330 (2.8)	
	No	3533	23.1	205 (5.8)		119 (3.4)	
	**Parental understanding**				0.004		0.101
	Yes	11,542	75.7	458 (4.0)		326 (2.8)	
	No	3703	24.3	188 (5.1)		124 (3.4)	
	**Parental monitoring**				0.006		< 0.001
	Yes	11,652	76.3	469 (4.0)		309 (2.7)	
	No	3625	23.7	184 (5.1)		139 (3.5)	
	**Parental intrusion of privacy**				< 0.001		< 0.001
	Yes	8248	45.8	388 (5.6)		283 (4.1)	
	No	8248	54.2	260 (3.2)		160 (1.9)	
	**Parental tobacco use**				< 0.001		< 0.001
	Yes	2354	15.3	282 (12.0)		186 (7.9)	
	No	13,020	84.7	369 (2.8)		265 (2.0)	

### Factors associated with cannabis and amphetamine use among school-going adolescents in across sub-Saharan

The results from the multivariate logistic regression analysis of determinants of cannabis and amphetamine use among school-going adolescents are shown in Table 2. In terms of *demographic factors*, females were less likely to use cannabis [AOR = 0.41, 95% CI = 0.31, 0.55] and amphetamine [AOR = 0.64, 95% CI = 0.64, 95% CI = 0.45,0.90] compared to males. The odds of past-month cannabis use were higher among adolescents aged 15 years [AOR = 2.26, 95% CI = 1.08, 4.70], 16 years [AOR = 2.54, 95% CI = 1.22, 5.31], and 17 years [AOR = 2.48, 95% CI = 1.14, 5.40].

With reference to *mental health factors*, participants who reported they have had suicidal ideation where less likely to report past-month cannabis use. However, adolescents who had attempted suicide had higher odds of both past-month cannabis use [AOR = 2.21, 95% CI1.57, 3.09] and amphetamine use [AOR = 3.65, 95% CI = 2.49, 5.36]. For *lifestyle factors*, we found that school-going adolescents who reported they smoked cigarette were more likely to report cannabis use in the past month [AOR = 10.38, 95% CI = 7.77, 13.87] and amphetamine [AOR = 3.84, 95% CI = 2.58, 5.71]. Additionally, adolescents who reported alcohol use in the past month had higher odds of using both cannabis [AOR = 2.82, 95% CI = 2.01, 3.97] and amphetamine [AOR = 2.14, 95% CI = 1.36, 3.35]. We also found that participants who reported lifetime drunkenness had higher odds of using cannabis [AOR = 2.64, 95% CI = 1.89, 3.67] and amphetamine [AOR = 3.16, 95% CI = 2.00, 4.96]. Leisure-time sedentary behaviour among school-going adolescents was only associated with higher odds of past-month cannabis use [AOR = 1.30, 95% CI = 1.00, 1.70].

For *school-level factors*, the odds of cannabis use [AOR = 1.96, 95% CI = 1.51, 2.55] and amphetamine use [AOR = 2.59, 95% CI = 1.84, 3.66] was higher among school-going adolescents who were truant. Adolescents who reported they were bullied were more likely to use cannabis [AOR = 1.42, 95% CI = 1.08, 1.86] and amphetamine [AOR = 1.71, 95% CI = 1.20, 2.44]. However, receiving social support in school was a protective factor against the use of amphetamine use [AOR = 0.64, 95% CI = 0.43, 0.96]. *Family-level factors* such as parental tobacco use was associated with increasing odds of both past-month cannabis use [AOR = 2.12, 95% CI = 1.62, 2.78] and lifetime amphetamine use [AOR = 1.63, 95% CI = 1.13, 2.37]. However, being monitored by parents was a protective factor against the use of amphetamine [AOR = 0.64, 95% CI = 0.43, 0.94].

School going adolescents from Ghana [AOR = 6.26, 95% CI = 2.40, 16.3], Liberia [AOR = 6.05, 95% CI = 2.39, 15.3], Mauritius [AOR = 4.39, 95% CI = 1.97, 9.80], Namibia [AOR = 3.31, 95% CI = 1.44, 7.14], Seychelles [AOR = 4.03, 95% CI = 1.79, 9.06], and Tanzania [AOR = 3.54, 95% CI = 1.38, 9.10] had higher odds of cannabis use. The odds of amphetamine use were higher among adolescents from Ghana [AOR = 3.07, 95% CI = 1.37, 6.88] but lesser among adolescents from Mauritius [AOR = 0.43, 95% CI = 0.20, 0.94] and Seychelles [AOR = 0.32, 95% CI = 0.14, 0.70].

**Table 2:** Multivariate logistic regression predicting risk and protective factors for cannabis and amphetamine use

Study Variables		Past-month cannabis use			Lifetime Amphetamine use		
		*β*	AOR [95% CI]	*p*-value	*β*	AOR [95% CI]	*p*-value
***Demographics***							
***Gender***				**< 0.001**			**0.010**
	Male		1			1	
	Female	-0.039	0.41 [0.31, 0.55]		-0.018	0.64 [0.45, 0.90]	
***Age***							
	12 years		1			1	
	13 years	0.000	1.47 [0.67, 3.25]	0.335	-0.009	0.98 [0.41, 2.33]	0.965
	14 years	-0.003	1.46 [0.68, 3.15]	0.335	-0.028	0.49 [0.20, 1.22]	0.126
	15 years	0.017	2.26 [1.08, 4.70]	**0.030**	-0.017	0.86 [0.38, 1.96]	0.729
	16 years	0.021	2.54 [1.22, 5.31]	**0.013**	-0.027	0.73 [0.32, 1.71]	0.417
	17 years	0.018	2.48 [1.14, 5.40]	**0.022**	-0.157	0.85 [0.36, 2.01]	0.713
***Mental health factors***							
	Anxiety	-0.008	1.16 [0.86, 1.56]	0.346	-0.000	1.17 [0.78, 1.74]	0.442
	Loneliness	-0.016	0.94 [0.70, 1.27]	0.700	-0.015	0.82 [0.56, 1.20]	0.298
	Suicidal ideation	-0.027	0.63 [0.44, 0.89]	**0.009**	-0.016	0.78 [0.52, 1.17]	0.232
	Suicidal attempt	0.055	2.21 [1.57. 3.09]	**< 0.001**	0.102	3.65 [2.49, 5.36]	**< 0.001**
***Lifestyle factors***							
	Cigarette smoking	0.335	10.38 [7.77, 13.87]	**< 0.001**	0.132	3.84 [2.58, 5.71]	**< 0.001**
	Past-month alcohol use	0.061	2.82 [2.01, 3.97]	**< 0.001**	0.040	2.14 [1.36, 3.35]	**0.001**
	Lifetime drunkenness	0.087	2.64 [1.89, 3.67]	**< 0.001**	0.079	3.16 [2.00, 4.96]	**< 0.001**
	Leisure-time sedentary behaviour	0.019	1.30 [1.00, 1.70]	**0.048**	0.021	1.41 [0.99, 1.99]	0.054
***School-level factors***							
	Truancy	0.058	1.96 [1.51, 2.55]	**< 0.001**	0.063	2.59 [1.84, 3.66]	**< 0.001**
	Bullying victimization	0.010	1.42 [1.08, 1.86]	**0.013**	0.029	1.71 [1.20, 2.44]	**0.003**
	Social support at school	-0.008	0.77 [0.54, 1.11]	0.160	-0.020	0.64 [0.43, 0.96]	**0.032**
***Interpersonal-level factors***							
	Number of close friends	0.001	0.96 [0.60, 1.52]	0.848	-0.013	0.62 [0.39, 1.00]	0.052
	Physical fight	0.016	1.17 [0.89, 1.54]	0.250	0.016	1.26 [0.89, 1.80]	0.195
***Family-level factors***							
	Parental supervision	-0.008	0.85 [0.63, 1.13]	0.266	0.015	1.22 [0.82, 1.82]	0.320
	Parental understanding	-0.003	0.98 [0.71, 1.33]	0.876	0.004	1.10 [0.73, 1.65]	0.646
	Parental monitoring	0.000	0.96 [0.70, 1.31]	0.785	-0.019	0.64 [0.43, 0.94]	**0.022**
	Parental intrusion of privacy	0.009	1.12 [0.85, 1.46]	0.423	0.019	1.36 [0.96, 1.93]	0.087
	Parental tobacco use	0.058	2.12 [1.62, 2.78]	**< 0.001**	0.035	1.63 [1.13, 2.37]	**0.010**
***Country***							
	Benin		1			1	
	Ghana	0.037	6.26 [2.40, 16.3]	**< 0.001**	0.033	3.07 [1.37, 6.88]	**0.006**
	Liberia	0.039	6.05 [2.39, 15.3]	**< 0.001**	0.006	1.12 [0.47, 2.70]	0.799
	Mauritius	0.041	4.39 [1.97, 9.80]	**< 0.001**	-0.028	0.43 [0.20, 0.94]	**0.035**
	Mozambique	0.021	2.59 [0.90, 7.42]	0.077	-0.004	0.73 [0.25, 2.16]	0.570
	Namibia	0.034	3.31 [1.44, 7.14]	**0.004**	0.031	1.39 [0.72, 2.69]	0.329
	Seychelles	0.027	4.03 [1.79, 9.06]	**0.001**	-0.055	0.32 [0.14, 0.70]	**0.005**
	Tanzania	0.047	3.54 [1.38, 9.10]	**0.009**	0.011	1.26 [0.55, 2.89]	0.579
Cox & Snell *R*^2^			0.105			0.048	
Nagelkerke *R*^2^			0.445			0.329	
Hosmer–Lemeshow GOF test (sig.)			8.25 (0.4094)			7.03 (0.5334)	
Overall % correctly classified			97.14%			98.52%	

*Note*. AOR = adjusted odds ratio; CI = Confidence Interval; statistically significant results are in boldface

## Discussion

### Summary of key findings

In this study, we examine the prevalence estimate and describe the correlates of cannabis and amphetamine use among school-going adolescents in eight sub-Saharan African countries (SSA). The overall prevalence estimates of past-month cannabis use and lifetime amphetamine use among school-going adolescents in the eight SSA countries was 4.39% and 3.05% respectively. These prevalence estimates for both past-month cannabis use and lifetime amphetamine were higher among males than females. In the multivariable logistic regression analysis, demographic characteristics (age and male gender), mental health factors (suicide ideation and attempt), lifestyle factors (cigarette smoking, past-month alcohol use, lifetime drunkenness and leisure-time sedentary behaviour) and school level factors (truancy and bullying victimisation showed strong associations with increased odds of both past-month cannabis use and lifetime amphetamine use. Social support at school was associated with increased odds for lifetime amphetamine, while parental monitoring decreases the odds for lifetime amphetamine use. It was also observed that parental tobacco use was associated with increased odds of both past-month cannabis use and lifetime amphetamine use.

### Prevalence of cannabis and amphetamine use

In this study we found 4.39% and 3.05% as the overall prevalence estimates of past-month cannabis use and lifetime amphetamine use respectively among school-going adolescents in the eight SSA countries. The higher prevalence rate of cannabis use compared to lifetime amphetamine is not surprising because within the literature, cannabis has comparatively low selling price, is locally sourced, early-age initiation, and low quit rate [1–3]. Additionally, social factors such as poverty, unemployment, and unfavorable social conditions also favour use, particularly among an increasing youth population within SSA [1, 2, 33]. The prevalence estimate of 4.39% for cannabis use is lower than what has been reported in other countries in SSA such as Ghana (5.3%) [7], 5.3% and 4.4% as prevalence estimates in Namibia and Swaziland respectively [12] and Nigeria (4.4%) [34] but relatively higher than the prevalence of 4.0% reported in Morocco [34]. Our prevalence estimate of 4.39% for past month cannabis use as reported in this study is similar to other rates reported in previous studies and reports in Nigeria and Mauritius [12, 34]. Similarly, the lifetime prevalence estimate of 3.05% for amphetamine use was lower than the 5.6-7.1% reported in other countries within SSA [7–9, 34]. The variations in the prevalence estimates for both past-month cannabis use and lifetime amphetamine use could be attributable to distal, familial, and contextual factors (e.g., easy access to drugs and the lack of enforcement and or absence of drug policies) within SSA [1, 2, 35]. It is also possible the variations in the reported prevalence rates could be due to the different study designs adopted, the population involved and the time-specific period in which these studies were conducted within each country.

While Seychelles had the highest level of cannabis use, school-going adolescents in Namibia reported the highest prevalence of amphetamine use. For Seychelles, the high prevalence of cannabis is not surprising, as the country has been reported to be suffering from a drug epidemic of huge proportions [33, 36, 37]. The high prevalence rate of amphetamine use in Namibia could be attributed to the fact that Namibia is one of the countries within the sub-region with highest prevalence alcohol dependency and other related drugs including amphetamine [38]. Although the prevalence estimates of both cannabis and lifetime amphetamine use were relatively lower as reported in this study, its adverse effect on young people calls for the development of contextual relevant and multi-sectoral intervention and school-based prevention programmes that could target school-going adolescents who may be at risk of misusing these illicit drugs.

### Factors associated with cannabis and amphetamine use

At the personal level, both age and gender were associated with both cannabis and amphetamine use among school-going adolescents. Specifically, females were less likely to use cannabis and amphetamine compared to males; and the odds of past-month cannabis use increases with age. These findings confirm what has been reported in previous studies within SSA among adolescents and young adults in Ghana [11], Mauritius, Namibia, and Swaziland [12], Nigeria [34], and Zambia [13] but contradicts an earlier study in Ghana which did not find any age and gender differences in both cannabis and amphetamine use [7]. Generally, these gender differences could be attributed to the fact that boys engage in more risky behaviours including substance use than females; and these are supported gendered social norms and conservative cultures within SSA. Again, developmentally, the transition from childhood to adulthood predisposes young people to increased vulnerability to adverse health risk behaviours [2], which could account for why the odds of past-month cannabis use increases with age.

At the personal level factors, it was found that mental health factors such as suicidal ideation was negatively associated with past-month cannabis use, but suicide attempt was associated with higher odds of both past-month cannabis use and amphetamine use. The positive relationship between suicide attempt and cannabis and amphetamine use is not surprising as previous studies had established that generally substance increases to the odds of acute life-threatening behaviours including suicide [9, 12, 14, 15]. It has globally been acknowledged that both cannabis and amphetamine have mind-altering compounds (like dopamine) that affect the brain and can potentially worsen the symptoms of any mental disorders including schizophrenia and other forms of psychoses [1, 2]. Indeed, a recent systematic review and meta-analysis had showed that cannabis use increased the risk of suicide attempt, suicidal ideation, and suicide planning in young individuals of 11–21 years of age [39]. Cannabis use generally impairs judgement and cognitive functions, and potentially complicates the natural course of loneliness, anxiety, and depression – which in turn elevate the risk for suicidal thoughts and behaviour among adolescents [10, 39–41]. However, a previous study in Ghana did not find any relationship between mental health factors and both cannabis use and amphetamine use [7]. Furthermore, the negative relationship between suicidal ideation and past-month cannabis use contradicts previous studies and calls for further studies that involve the use of longitudinal studies and well thought of qualitative studies to enhance our understanding of the relationship between alcohol use and suicidal behaviour.

Our findings further showed that at the personal level, lifestyle factors (cigarette smoking, past month alcohol use, lifetime drunkenness and leisure-time sedentary behaviour) were associated with increased odds of both past-month cannabis and lifetime amphetamine use. Global and regional systematic review [33, 36, 39] and nearly all the primary studies drawing GSHS data across the African region [9, 12, 15, 28] confirms the possible existence of clustering or concurrent health-compromising behaviours among school-going adolescent in SSA. Our results also affirms the observation that young adults within sub-Saharan Africa continue to face multiple challenges that predispose them to several health risk behaviours [42, 43]. Furthermore, the existence of clustering or concurrent health-compromising behaviours underscore the need for the development of poly-substance use interventions among adolescents.

In our study, school-level factors such as truancy and bullying victimisation were found to increase the odds of both cannabis and amphetamine use. However, receiving social support in school was a protective factor against the use of amphetamine. The relationship between illicit drug use and truancy among school-going students have been reported by previous studies [7, 12, 13]. This relationship should be interpreted cautiously given that this relationship has been shown to be mediated by biological (e.g., cognitive function), behavioural (e.g., school attendance) and emotional or mental health factors (e.g., poor social connection) [28, 44, 45]. The positive relationship between bullying victimisation and both cannabis and amphetamine use is replete within the literature [2, 7, 12, 13] and could possibly be due to the fact that adolescents who are bullied or have prior victimisation experiences may resort to illicit substance use as a coping strategy to deal with the challenges associated with bullying within the school context. For example, it has been established that adolescents who experience bullying victimisation are at risk of developing both internalising and externalising problems including substance abuse and misuse [2, 46, 47]. With high level of bullying victimisation reported among in-school adolescents within the sub-region [48] and the fact that there are no anti-bullying policies within high schools [49], provides an important pathway and information that can be considered in the development of school-based mental health policy and gatekeeping training programmes that addresses bullying victimisation among adolescents. We also found social support at school (defined as how helpful and kind most of the students in your school were) to be associated with reduced odds of amphetamine use. This confirms previous studies that have reported social support to be protective of amphetamine use in school-going adolescents [7, 12, 17]. These findings suggest that good supportive friendships among adolescents may lead to development of key positive health outcomes.

As reported in previous studies [7, 12, 18], we also found that family-level factors such as parental tobacco use was associated with increasing odds of both past-month cannabis use and lifetime amphetamine use. It is possible that the behaviour of parents who smoke tobacco may send a signal to their children that such behaviour is acceptable for them to imitate. However, being monitored by parents was a protective factor against the use of amphetamine. The negative relationship between parental monitoring and adverse health behaviour including illicit drug use have been documented in previous studies in Africa [7–9, 12] and a recent systematic review [19]. It is plausible that parents who engaged meaningfully with their adolescent child have better knowledge about where and with whom their adolescent child associate, and (co-)create rules that limit the engagement in adverse health behaviours including illicit drug use.

### Strengths and limitations of the study

The findings of this study has some limitations that should be noted when interpreting them. Firstly, as the WHO-GSHS data were collected using a one-off cross-sectional design, all the examined variables were measured at one point in time. Thus, assessment of causation and temporal relationship among the outcomes and exposure variables becomes a challenge. Secondly, the assessment of the variables in the study was based on a self-report, which are prone to recall bias and social desirability effects since the use of both cannabis and amphetamine use among underage persons maybe prohibited in the various SSA countries. Thirdly, it should also be noted that the study included only students who were present at school on the day of the survey; besides plausible variations in attendance rates, and that out-of-school going adolescents were not included. Finally, some possible behavioural and psychological factors that have been shown to be associated with both cannabis and amphetamine use were not considered since they were not available in the GSHS datasets. Taken together, these factors might have presented as potential sources of bias to this analysis, including possible underestimation of the prevalence of illicit drug use. Notwithstanding these limitations, this is one of the first studies to have a nationally representative data from eight countries in SSA to advance our knowledge of the prevalence estimates and factor associated with both cannabis and amphetamine use among in-school adolescents. Additionally, the use of a large sample obtained using a multi-sage systematic random sampling allows for generazability of our findings to other similar populations.

## Conclusion

This study examined prevalence estimates and described the correlates of cannabis and amphetamine use among school-going adolescents in eight sub-Saharan African countries (SSA) – Benin, Ghana, and Liberia, Mauritius, Mozambique, Namibia, Seychelles, and Tanzania. Our results found a relatively low overall prevalence estimates of past-month cannabis use and lifetime amphetamine use among school-going adolescents. We also found that both past-month cannabis use and lifetime amphetamine were associated with various multi-level factors including sociodemographic, family involvement, mental health, and school environment factors. These findings of this study support existing evidence and underscore the need for these eight countries in SSA to develop contextual and multi-sectoral intervention and school-based prevention programmes that could target school-going adolescents who may be at risk of misusing these illicit drugs.

### Electronic supplementary material

Below is the link to the electronic supplementary material.

Supplementary Material 1 e-Table 1. Coding of demographic variables and exposure factors included study, and missing data

## Data Availability

The datasets used and/or analysed during the current study are freely available from the WHO website: https://extranet.who.int/ncdsmicrodata/index.php/catalog/GSHS. The data was not collected by the authors and others will be able to access the data just as we did.

## Abbreviations

CDC: Centres for Disease Prevention
GSHS: Global School-based Student Health Survey
SSA: sub-Saharan Africa
WHO: World Health Organization
