# Supplementary material for: Cannabis and amphetamine use among school-going adolescents in sub-Saharan Africa: a multi-country analysis of prevalence and associated factors
Source: BMC Psychiatry. 2023 Oct 24;23:778. doi: 10.1186/s12888-023-05283-w (PMC10599041; doi:10.1186/s12888-023-05283-w)
Supplement: Supplementary file 1 — Supplementary Material 1 e-Table 1. Coding of demographic variables and exposure factors included study, and missing data [file 12888_2023_5283_MOESM1_ESM.doc]

**Supplementary Material**

e-Table 1. Coding of demographic variables and exposure factors included study, and missing data

| **Variable** | **Survey question** | **Response coding** | **Observation / Missing (%)** |
| --- | --- | --- | --- |
| **Outcome variable** |  |  |  |
| Past-month cannabis/marijuana use | During the past 30 days, how many times have you used marijuana | None (0)  1 or more (1) | N = 15, 553/ 0 (0%) |
| Lifetime amphetamine use | During your life, how many times have you used amphetamine or methamphetamine (also called ice or yellow) | None = 0  1 or more times =1 | N = 15, 553/ 0 (0%) |
| **Socio-demographics** |  |  |  |
| Gender | What is your sex | Male (0)  Female (1) | N=15,553/0 (0%) |
| Age | How old are you? | 12–17 years (continuous) | N=15,553/0 (0%) |
| **Mental health factors** |  |  |  |
| Anxiety | During the past 12 months, how often have you been so worried about something that you could not sleep at night? | Never (0)  Rarely–always (1) | N=15,351/202 (1.3%) |
| Loneliness | During the past 12 months, how often have you felt lonely? | Never (0)  Rarely–always (1) | N= 15,389/164 (1.1%) |
| Suicide ideation | During the past 12 months, did you ever seriously consider attempting suicide? | No (0)  Yes (1) | N=15,206/347 (2.2%) |
| Suicide attempt | During the past 12 months, how many times did you actually attempt suicide? | 0 times (0)  1 time to 6 or more times (1) | N=15,313/240 (1.5%) |
| **Lifestyle factors** |  |  |  |
|  |  |  |  |
| Cigarette smoking | During the past 30 days, on how many days did you smoke cigarettes? | 0 days=0  1or 2 days to All 30 days=1 | N=15,279/274 (1.8%) |
| Past month alcohol use | During the past 30 days, on how many days did you have at least one drink containing alcohol? | 0 days = 0  1or 2 days to All 30 days=1 | N=14,782/771 (5.0%) |
| Lifetime drunkenness | During your life, how many times did you drink so much alcohol that you were really drunk? | 0 times = 0  1or 2 times to 10 or more times=1 | N=15014/539 (3.5%) |
| Leisure-time sedentary behaviour | How much time do you spend during a typical or usual day sitting and watching television, playing computer games, talking with friends, or doing other sitting activities such as playing cards, dominos, chess, and scrabble. | Less than 1 hour per day/1 to 2 hours per day=0  3-4 hours to more than 8 hours per day=1 | N=15246/307 (2.0%) |
|  |  |  |  |
|  |  |  |  |
| **Family-level factors** |  |  |  |
| Parental understanding | During the past 30 days, how often did your parents or guardians understand your problems and worries? | Never (0)  Rarely–always (1) | N=15,245/308 (2.0%) |
| Parental monitoring | During the past 30 days, how often did your parents or guardians really know what you were doing with your free time? | Never (0)  Rarely–always (1) | N=15,2777/276 (1.8%) |
| Parental supervision | During the past 30 days, how often did your parents or guardians check to see if your homework was done? | Never (0)  Rarely–always (1) | N=15,283/270 (1.7%) |
| Intrusion of privacy by parents | During the past 30 days, how often did your parents or guardians go through your things without your approval | Never (0)  Rarely–always (1) | N=15,220/333 (2.1%) |
| Parental tobacco use | Which of your parents or guardians use any form of tobacco? | Neither/I do not know=0  My father or male guardian/My mother or female guardian/Both=1 | N=15,374/179 (1.2%) |
| **School-level factors** |  |  |  |
| Truancy | During the past 30 days, how many days did you miss classes or school without permission? | None (0)  1 or more (1) | N=15,256/297 (1.9%) |
| Bullying victimisation | During the past 30 days, how many days were you bullied? | Never (0)  1 or more (1) | N=14,438/1115 (7.2%) |
| Social support at school | During the past 30 days, how often were most of the students in your school kind and helpful? | Never (0)  Rarely–always (1) | N=15281/272 (1.8%) |
| **Interpersonal factors** |  |  |  |
| Number of close friends | How many close friends do you have? | None (0)  1 or more (1) | 15,296/257 (1.7%) |
| Physical fights | During the past 12 months, how many times were you in a physical fight? | None (0)  1 or more (1) | 16,424/129 (0.8) |
